# DOE-Based Optimization of Dietary Fiber Extraction Process and Bioactivity Evaluation of Plum (*Prunus salicina* Lindl.) Processing By-Products

**DOI:** 10.3390/foods15071199

**Published:** 2026-04-02

**Authors:** Juan Chen, Xueting Zhang, Xin Hu, Yan Wen, Dongyan Huang, Xiaoyu Wen, Guiqun Song, Qi Yuan, Xudong Liu

**Affiliations:** 1 School of Food Engineering, Moutai Institute, Renhuai, Zunyi 564507, China; chenjuan602@163.com (J.C.); e2822751693@163.com (X.Z.); 15084433356@163.com (X.H.); 15599266870@163.com (Y.W.); 15685330201@163.com (D.H.); 19184457596@163.com (X.W.); 18385500841@163.com (G.S.); 18311667945@163.com (Q.Y.); 2 Engineering Research Center for Development and Comprehensive Utilization of Guizhou Characteristic Food Resources, Zunyi 564507, China

**Keywords:** plum pomace, design of experiments (DOE), soluble dietary fiber (SDF), insoluble dietary fiber (IDF), functional modification, extraction optimization

## Abstract

Plum pomace (PP), a key by-product of plum juice processing, is a rich yet underutilized source of dietary fiber. However, its high-value exploitation is severely limited by the lack of efficient extraction and modification technologies. This study optimized the extraction of soluble dietary fiber (SDF) and insoluble dietary fiber (IDF) from plum pomace (PP) via Design of Experiments (DOE), and evaluated their modification effects. Alkaline extraction was screened as the optimal method for IDF, and orthogonal experiments determined the optimal conditions: solid-to-liquid ratio 1:20 g/mL, 14 g/L NaOH, 60 °C, and 80 min, achieving a high extraction yield of 62.18%. For SDF, enzymatic extraction was superior, and response surface methodology (RSM) optimized the process to a solid-to-liquid ratio of 1:15.5, 1.0% enzyme dosage, 61.5 °C, and 92 min, with a yield of 29.3%. Physical, chemical, and biological modifications all significantly enhanced SDF’s water/oil-holding capacity, cholesterol/glucose adsorption capacity, and cation exchange capacity. Biologically modified SDF showed the most significant enhancement, with WHC of 5.58 ± 0.05 g/g, OHC of 4.38 g/g, CAC of 7.68 mg/g, and CEC of 3.28 mmol/g. These results provide technical support for the high-value utilization of PP and lay a foundation for its application in functional foods and nutraceuticals.

## 1. Introduction

Plum (*Prunus salicina* Lindl.) is a nutrient-dense fruit, valued for its abundant bioactive compounds and associated health benefits [1,2]. It is widely processed into juice, generating significant by-products. According to the FAO 2024 data, global production of plums and sloes reached approximately 12.69 million metric tons. China, as the core production region, contributed 6.91 million metric tons, accounting for over 54% of the world’s total output. Plum pomace (PP), a by-product generated during processing, typically accounts for 20–30% of fresh fruit weight, equivalent to an estimated 2.5–3.8 million metric tons globally per year. It possesses considerable amounts of valuable bioactive components, including flavonoids, phenolic acids, and anthocyanins [3,4], and is also a good source of dietary fiber [5]. However, this by-product is often discarded, leading to substantial resource waste and potential environmental pollution [6,7]. As a promising source of high-quality natural dietary fiber (DF) [7,8,9], PP is of great potential for high-value utilization, while DF is classified as the seventh major nutrient for its vital roles in gut health promotion, blood glucose and lipid regulation, and cancer risk reduction [10]. Based on water solubility, DF is classified into soluble (SDF) and insoluble (IDF) fractions [11]. DF has been widely applied in the food and nutraceutical industries, with IDF showing superior water-holding capacity (WHC) and oil-holding capacity (OHC) [12], while SDF is known for its excellent adsorption properties and structural malleability for functional modification [13,14]. Thus, developing efficient extraction and modification technologies for PP-derived DF is of significant scientific and practical value [15].

Acid, alkaline, and enzymatic methods are the most commonly used approaches for DF extraction: acid and alkaline treatments are operationally simple but may cause fiber structural degradation [16], while enzymatic extraction features high selectivity and mild conditions that better preserve the native fiber structure. Additionally, physical, chemical, and biological modification methods can further optimize the physicochemical and functional properties of DFs [14], with physical modification enhancing WHC and swelling capacity (SW), chemical modification improving adsorption capabilities, and biological modification boosting bioactivity via microbial fermentation [12]. Design of Experiments (DOE) is a statistically based methodology that systematically investigates multi-factor effects and their interactions, which avoids experimental errors of the traditional one-factor-at-a-time (OFAT) approach and improves experimental efficiency, making it widely applicable in food science and industrial production [8]. However, to our knowledge, no studies have yet employed DOE to optimize the extraction process of DFs from PP.

This study aimed to (1) subsequently optimize the extraction of SDF and IDF from plum pomace using DOE methodologies (orthogonal design for IDF, response surface methodology for SDF), after comparing acid, alkaline, and enzymatic methods; (2) apply and compare physical, chemical, and biological modifications to the extracted SDF; and (3) comprehensively evaluate the modified SDF based on six key techno-functional indicators (WHC, OHC, SC, CAC, GAC, CEC) to identify the most promising modification strategy for food-grade applications. The innovations of this study are as follows: (1) for the first time, DOE-based orthogonal test and RSM are applied to simultaneously optimize the extraction of SDF and IDF from Guizhou-sourced PP, clarifying the key influencing factors and their interactions to achieve high yields; (2) a comprehensive evaluation system covering six functional indicators (WHC, OHC, SW, CAC, GAC, CEC) is established to compare the modification effects of physical, chemical, and biological methods, identifying the most suitable food-grade modification technology for PP-SDF; and (3) this study links local plum industry waste with high-value DF production, providing targeted technical support for the utilization of agricultural by-products in Guizhou. This study is expected to provide a theoretical basis for the efficient extraction and high-value utilization of PP-derived DF, as well as data support for its application in functional foods and nutraceuticals.

## 2. Materials and Methods

### 2.1. Materials

*Prunus salicina* purchased from local markets was washed, pitted, and juiced. The residual pomace was dried in an oven at 60 °C for 36 h to a final moisture content of less than 8%, then ground and sieved through a 40-mesh screen (particle size < 425 μm) to obtain PP powder. Papain and α-amylase were purchased from Aladdin Biochemical Technology Co., Ltd. (Shanghai, China), with enzyme activities of 800 U/mg and 35 U/mg, respectively. Cholesterol standard, 3,5-dinitrosalicylic acid (DNS), phenolphthalein indicator, and potassium sodium tartrate tetrahydrate were obtained from Sinopharm Chemical Reagent Co., Ltd. (Shanghai, China). *Penicillium* sp. YZ-1 was provided by the laboratory of Nanchang University (Nanchang, China). Ultrapure water was used throughout the experiments, and all other chemicals were of analytical grade.

### 2.2. PP IDF

#### 2.2.1. Extraction Method Investigation

The enzymatic method was adapted from Zhao [17] and Jiang [18] with slight modifications. Accurately weigh 10.0 g of PP powder, control the addition of composite enzyme at 0.4%, and adjust the solid–liquid ratio to 1:15 g/mL. The pH of the suspension was adjusted to 6.0, followed by extraction at 60 °C for 60 min. The mixture was filtered through a quantitative filter paper (Hawach BIO-40), and the residue was dried to constant weight to obtain IDF.

The acid method was adapted from the methods of Berktas [19] and Wen [20] with slight modifications. Accurately weigh 10.0 g of plum fruit residue powder, extract at an acid concentration of 0.15 mol/L and a solid–liquid ratio of 1:15 g/mL for 60 min at 60 °C, and the pH was neutralized with NaOH solution. The mixture was filtered, and the precipitate was dried to constant weight to obtain acid-extracted PP IDF.

The alkaline method was adapted from Kumari [21] and Xie [22] with slight modifications. Accurately weigh 10.0 g of plum fruit residue powder, add a 1:15 g/mL sodium hydroxide solution with a concentration of 8 g/mL, extract at 60 °C for 60 min, and then adjust the pH to approximately 7.0 with HCl. filtered, and dried the precipitate to constant weight to obtain alkaline PP IDF.

The extraction rate of IDF was calculated as follows:

(1) $$P\;=\;\frac{m}{M}\;\times \;100\%$$

where P = extraction rate of IDF, expressed as a percentage (%); m = mass of the obtained IDF, with the unit of gram (g); M = mass of the weighed plum pomace powder sample, with the unit of gram (g).

#### 2.2.2. Optimization of Extraction Method Through OFAT

Determination of solid-to-liquid ratio. Weigh the same quality of PP powder, select an extraction time of 60 min, a sodium hydroxide concentration of 5 g/L, an extraction temperature of 80 °C, and compare the effects of solid–liquid ratios of 1:10, 1:15, 1:20, 1:25, 1:30 g/mL on IDF yield.

Determination of extraction temperature. Weigh the same quality of PP powder, select a solid–liquid ratio of 1:20 g/mL, a sodium hydroxide concentration of 5 g/L, and an extraction time of 60 min to compare the effects of extraction temperatures of 50, 60, 70, 80, 90 °C on the IDF yield.

Determination of sodium hydroxide concentration. Weigh the same quality of PP powder. Set the liquid-to-material ratio to 1:20 g/mL, temperature to 40 °C, and alkaline hydrolysis time to 60 min. Then, equally weigh the plum fruit residue powder and compare the effects of sodium hydroxide concentrations of 2, 5, 8, 11, and 14 g/L on the IDF yield.

Determination of extraction time. Weigh the same quality of PP powder, select a solid–liquid ratio of 1:20 g/mL, sodium hydroxide concentration of 5 g/L, and extract at 80 °C for 20, 40, 60, 80, 100 min, respectively. Compare the effect of different extraction times on the IDF yield.

#### 2.2.3. Optimization Through Orthogonal Experimental Design

In order to obtain the optimal process parameters, based on single-factor experiments, extraction temperature (A), extraction time (B), sodium hydroxide concentration (C), and solid–liquid ratio (D) were selected as experimental factors. An L9 (3^4^) orthogonal experiment was designed to optimize the extraction process of IDF from PP, with the yield as the evaluation index. Factor and level design for the extraction process of IDF from PP are presented in Table 1.

### 2.3. PP SDF

#### 2.3.1. Extraction Method Investigation

Acid extraction. PP was first oven-dried, pulverized, and then passed through a 0.22 mm (60-mesh) sieve. A total of 10 g of sieved PP was weighed into a beaker and extracted with 1% (*w*/*v*) hydrochloric acid at 80 °C for 60 min at a solid-to-liquid ratio of 1:20 (g/mL). After extraction, the slurry was filtered through a quantitative filter paper (Hawach BIO-40); the filtrate was concentrated under vacuum to one-fifth of its original volume. Four volumes of 95% (*v*/*v*) ethanol were added to precipitate the SDF. The mixture was left overnight, then centrifuged at 6000× *g* for 20 min. The supernatant was discarded, and the pellet was dried at 60 °C to constant weight to obtain the SDF from PP [23].

Alkaline extraction. PP was oven-dried, pulverized, and sieved through a 0.22 mm (60-mesh) screen. A total of 10 g of screened PP was placed in a beaker and extracted with 1% (*w*/*v*) NaOH at 80 °C for 60 min at a solid-to-liquid ratio of 1:20 (g/mL). After extraction, the pH was adjusted to 4.5 with HCl, followed by vacuum filtration. The filtrate was concentrated to one-fifth of its original volume under reduced pressure, precipitated with four volumes of 95% ethanol, and left to stand overnight. The precipitate was collected by centrifugation at 6000× *g* for 20 min. The supernatant was removed, and the pellet was dried at 60 °C to constant weight to yield the SDF [22].

Enzymatic extraction. PP was oven-dried, ground, and sieved through a 0.22 mm (60-mesh) sieve. A total of 10 g of resulting powder was transferred to a beaker, suspended in distilled water at a 1:20 (g/mL) solid-to-liquid ratio, and supplemented with 1% (*w*/*w*) of an enzyme mixture (papain: α-amylase = 1:1). The pH of the suspension was adjusted to 6.0 using 0.1 M HCl or NaOH. The suspension was incubated at 80 °C for 1 h, after which enzymes were inactivated by boiling for 10 min. The mixture was filtered; the filtrate was concentrated under vacuum to one-fifth of its original volume. Four volumes of 95% ethanol were added to precipitate the SDF [24], which was left overnight, then centrifuged at 6000× *g* for 20 min. The supernatant was discarded, and the precipitate was dried at 60 °C to constant weight to obtain the SDF. 

#### 2.3.2. Optimization of Extraction Method Through OFAT

Solid-to-liquid ratio. The solid-to-liquid ratio varied at 1:10, 1:15, 1:20, 1:30 g/mL while keeping all other parameters constant. SDF was extracted enzymatically, and the yield was measured to identify the optimal level.

Extraction time. Extraction times of 30, 60, 90, 120, and 150 min were tested, with all other factors held constant. Enzymatic extraction was employed, and the SDF yield was determined to select the best time.

Extraction temperature. Extraction temperatures of 40, 50, 60, 70, and 80 °C were investigated under otherwise fixed conditions. Enzymatic extraction was performed, and the SDF yield was evaluated to establish the optimum temperature.

Enzyme dosage. Enzyme concentrations of 0.5%, 1.0%, 1.5%, 2.0%, and 2.5% (*w*/*w*, relative to pomace) were examined while other variables remained unchanged. The enzymatic extraction was carried out, and the SDF yield was measured to determine the optimal enzyme dosage.

#### 2.3.3. Response Surface Optimization of Extraction Conditions

Based on single-factor experiments, the solid-to-liquid ratio (g/mL), enzyme dosage (%), temperature (°C), and extraction time (min) were selected as independent variables, while the yield of SDF served as the response value for response surface optimization. A four-factor, three-level Box–Behnken design (BBD) was employed using Design-Expert (version 10.0.3) to establish the response surface experimental plan. The coded and actual levels of the variables are presented in Table 2.

### 2.4. Modification of SDF Form PP

#### 2.4.1. Physical Modification

SDF from PP (1.0 g) was dispersed in distilled water at a solid-to-liquid ratio of 1:30 (g/mL) and thoroughly stirred. The suspension was then treated in an autoclave at 0.1 MPa (which corresponds to 121 °C) for a duration of 20 min. After cooling to room temperature (approximately 25 °C), the mixture was centrifuged at 8000× *g* for 15 min, and the supernatant was collected. Four volumes of 95% (*v*/*v*) ethanol (relative to the supernatant volume) were added, and the solution was left at 4 °C overnight (for 12 h) for alcohol precipitation. The precipitate was collected by centrifugation at 8000× *g* for 15 min, dried at 60 °C to constant weight, and designated as high-temperature/pressure-modified SDF [25].

#### 2.4.2. Chemical Modification

SDF (60.0 g) was suspended in isopropanol at a solid-to-solvent ratio of 1:30 (*w*/*v*) and magnetically stirred for 30 min. With continuous stirring, 75.6 mL of 3.25 mol/L NaOH was added dropwise, and the reaction was maintained for 3 h. Afterward, the slurry was cooled to room temperature and neutralized with 3 mol/L HCl. The mixture was refrigerated at 4 °C overnight to allow precipitation. The precipitate was separated, dried, and ground to yield chemically modified SDF [26].

#### 2.4.3. Biological Modification

Biological modification was conducted using *Penicillium* sp. YZ-1, a cellulose-degrading fungus previously isolated from naturally moldy pomelo peel. This strain was selected for its demonstrated efficacy in enhancing the yield and functionality of soluble dietary fiber from similar pomaceous byproducts [27].

The preserved strain was first activated on potato dextrose agar (PDA) plates. For inoculum preparation, the activated culture was washed with sterile physiological saline (0.85% NaCl) to obtain a spore suspension, which was adjusted to a concentration of 1 × 10^7^ CFU/mL. This spore suspension served as the inoculum for the seed culture, prepared by transferring it into seed culture medium (PDB) at 5% (*v*/*v*) and incubating at 28 °C for 2 days.

For the main fermentation, 10.0 g of SDF was dispersed in distilled water. The SDF suspension was inoculated with the prepared seed culture at a rate of 12% (*v*/*v*). Fermentation proceeded at 28 °C for 7 days, with the mixture manually shaken twice daily.

Post-fermentation, the broth was centrifuged (8000× *g*, 20 min) to remove mycelia. The collected supernatant was heated at 90 °C for 15 min to terminate biological activity. The modified SDF was then recovered from the supernatant via alcohol precipitation (as per Section 2.4.1) and dried at 60 °C to constant weight.

### 2.5. Determination of DFs Activity in PP

#### 2.5.1. OHC Determination

Weigh 1.0 g (W_1_) of DF from PP into two separate 50 mL centrifuge tubes (W_0_). Add 30 mL of corn oil to each tube. The mixtures were vortexed for 1 min to ensure complete dispersion and then allowed to stand at room temperature (25 °C) for 24 h to reach equilibrium. Centrifuge at 5000× *g* for 10 min, decant the supernatant oil, and drain the tubes upside-down for 5 min. Immediately weigh the residue (W_2_). Perform three parallel determinations and take the average. Calculate the OHC using Equation (2) [28]:

(2) $$\mathrm{OHC}/(g\cdot g^{-1})=\frac{W_{2}-\;W_{1}}{W_{1}}$$

where W_2_ is the mass (g) of PP DF after oil absorption, and W_1_ is the initial mass (g) of PP DF.

#### 2.5.2. WHC Determination

Accurately weigh 1.0 g (W_3_) of DF from PP into two separate 50 mL centrifuge tubes. Add 40 mL of distilled water to each tube and allow the samples to stand at room temperature for 24 h. Centrifuge at 5000× *g* for 10 min, discard the supernatant, and drain the tubes for 5 min. Immediately weigh the remaining residue (W_4_). Perform three parallel determinations and calculate the mean value. The WHC is then calculated using Equation (3) [29]:

(3) $$\mathrm{WHC}/(g\cdot g^{-1})=\frac{W_{4}-\;W_{3}}{W_{3}}$$

where W_4_ is the mass of PP DF after water absorption, g; W_3_ is the initial mass of PP DF, g.

#### 2.5.3. SC Determination

Accurately weigh 1.0 g (M_1_) of DF from PP into two dry 15 mL graduated centrifuge tubes and record the initial volume (V_1_, mL). Add 10 mL of distilled water to each tube, vortex to mix thoroughly, then allow the samples to soak at room temperature for 24 h. After equilibration, gently tap the tubes to remove trapped air and read the final settled volume (V_2_, mL). Carry out three parallel determinations and use the mean values to calculate the SC according to Equation (4) [30]:

(4) $$\mathrm{SC}/(\mathrm{mL}\cdot g^{-1})=\frac{V_{2}\;-\;V_{1}}{M_{1}}$$

where V_2_ is the volume of PP DF after soaking, V_1_ is the initial volume of PP DF (mL), and M_1_ is the mass of PP DF (g).

#### 2.5.4. Cholesterol Adsorption Capacity (CAC) Determination

Cholesterol concentration was quantified by the O-phthaldialdehyde (OPA) method. Fresh egg yolk was diluted 1:9 (*w*/*v*) with distilled water and thoroughly homogenized. Exactly 2.0 g of DF from PP was weighed into separate Erlenmeyer flasks, followed by the addition of 50 g of the diluted yolk. The pH of each suspension was adjusted to 2.0 or 7.0 with 1 mol/L HCl or NaOH. Samples were shaken at 37 °C for 2 h and then centrifuged at 2000× *g* for 15 min. One milliliter of the supernatant was withdrawn, diluted five-fold with 90% (*v*/*v*) glacial acetic acid, and the absorbance was measured at 550 nm using a UV-Vis Spectrophotometer (UV-5200PC, Varioskan Flash, Shanghai Yuanxi Instrument Co., Ltd., Shanghai, China). The cholesterol content was calculated from a standard curve and expressed as mg cholesterol adsorbed per gram of DF. Carry out three parallel determinations and use the mean values to calculate the SC according to Equation (5) [31]:

(5) $$\mathrm{CAC}/(\mathrm{mg}\cdot g^{-1})=\frac{A_{1}-A_{2}}{M_{1}}$$

where A_1_, cholesterol content before adsorption by DF, mg/g; A_2_, cholesterol content after adsorption by DF, mg/g; M_1_, mass of PP DF, g.

#### 2.5.5. Glucose Adsorption Capacity (GAC) Determination

A total of 0.50 g of the dried sample (obtained from 2.5 g of PP DF pretreated with 200 mL of 85% ethanol at 80 °C for 15 min, followed by filtration and drying to constant weight at 60 °C) was mixed with 100 mL of 100 mmol/L glucose solution. The mixture was incubated at 37 °C with shaking for 6 h. After incubation, the mixture was centrifuged at 1700× *g* for 20 min. The supernatant was collected and diluted to a final volume of 100 mL. The residual glucose concentration in the diluted supernatant was determined using the DNS colorimetric method using a UV-Vis Spectrophotometer and calculating the GAC using Equation (6) [32]:

(6) $$\mathrm{GAC}/(\mathrm{mmol}\cdot g^{-1})=\frac{{(C}_{1}{-C}_{2})\;\times \;V}{m}$$

where C_1_, glucose concentration before adsorption by DF, mmol/L; C_2_, glucose concentration after adsorption by DF, mmol/L; V, constant volume, L; m, mass of PP DF, g.

#### 2.5.6. Cation Exchange Capacity (CEC) Determination

Accurately weigh 1.0 g of PP DF into separate 100 mL ground-glass Erlenmeyer flasks. Add 50 mL of 1 mol/L HCl, stopper the flasks, mix thoroughly, and allow the suspension to stand at room temperature for 24 h. Filter and collect the residue; transfer it to a 250 mL ground-glass Erlenmeyer flask and add 150 mL of 5 g/100 mL NaCl solution. Stir for 30 min to exchange the bound H^+^ ions, then titrate the liberated H^+^ with standardized NaOH (0.1 mol/L) using phenolphthalein as an indicator. Calculate the CEC according to Equation (7) [33]:

(7) $$\mathrm{CEC}/(\mathrm{mmol}\cdot g^{-1})=\frac{{(V}_{1}-{\;V}_{0})\;\times \;C}{m}$$

where V_0_, volume of NaOH standard solution consumed by the blank, mL; V_1_, volume of NaOH standard solution consumed by the sample, mL; C, exact concentration of the NaOH standard titrant, mol/L; m, mass of PP DF, g.

### 2.6. Statistical Analysis

All experiments were performed in triplicate, and data are presented as mean ± standard deviation (SD). One-way ANOVA with Duncan’s test (*p* < 0.05) was conducted using SPSS (version 21.0). RSM data were modeled and analyzed via ANOVA using Design-Expert (version 13) with center point replication.

## 3. Results and Discussion

### 3.1. Optimization of Extraction Conditions for IDF from PP

#### 3.1.1. Determination of the Extraction Method

**Figure 1:**
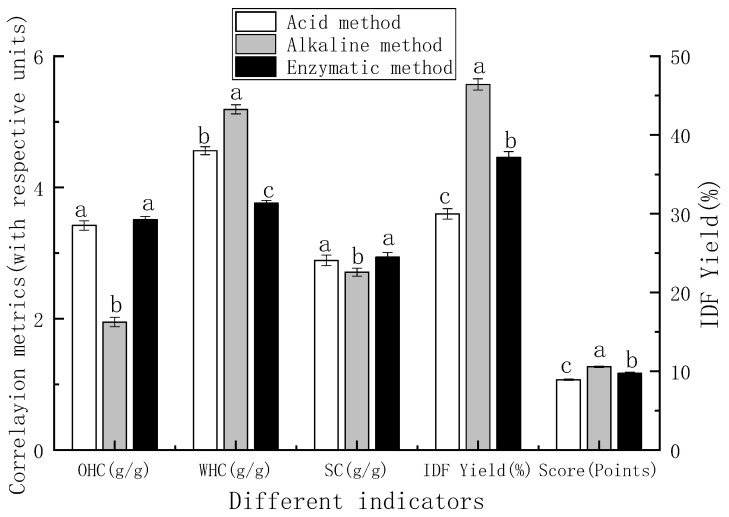
Comparison of different extraction methods for IDF. Note: The right vertical axis represents the values of extraction yield, while the left vertical axis represents those of OHC, WHC, SC, and the score. Different letters denote significant differences (*p* < 0.05).

The extraction yields, OHC, WHC, and SC obtained by acid, alkaline, and enzymatic methods were 29.98%, 3.420 g/g, 4.560 g/g, 2.888 mL/g; 46.42%, 1.950 g/g, 5.190 g/g, 2.710 mL/g; and 37.14%, 3.510 g/g, 3.761 g/g, 2.936 mL/g, respectively. Alkaline extraction exhibited the highest yield and WHC, whereas enzymatic extraction resulted in the highest OHC and SC. A multi-criteria comprehensive evaluation, using a normalization method, was then applied for scoring. As clearly illustrated in Figure 1, the alkaline method achieved the highest extraction yield and overall score for IDF from PP (the weight coefficients were assigned as follows: the extraction yield accounted for 0.8, while the WHC, OHC, and SC collectively accounted for the remaining 0.2) outperforming both enzymatic and acid methods. Moreover, alkaline extraction offers advantages such as simple operation and low cost; therefore, it was selected for further optimization.

In this study, it was found that the extraction yield of IDF from plum pomace by the alkaline method (46.42%) was significantly higher than that by the acidic method (29.98%) and the enzymatic method (37.14%), which is consistent with the research conclusion of Kumari et al. [21] on the extraction of IDF from pea hulls—alkaline conditions can effectively destroy the cell wall structure and promote the release of cellulose. However, too high alkali concentration (>14 g/L) will lead to lignin degradation, thereby reducing the yield, which is consistent with the “alkali concentration threshold effect” observed by Xie et al. [22] in the extraction of IDF from pomelo peel.

#### 3.1.2. Single Factor Optimization of Extraction Method for IDF

**Figure 2:**
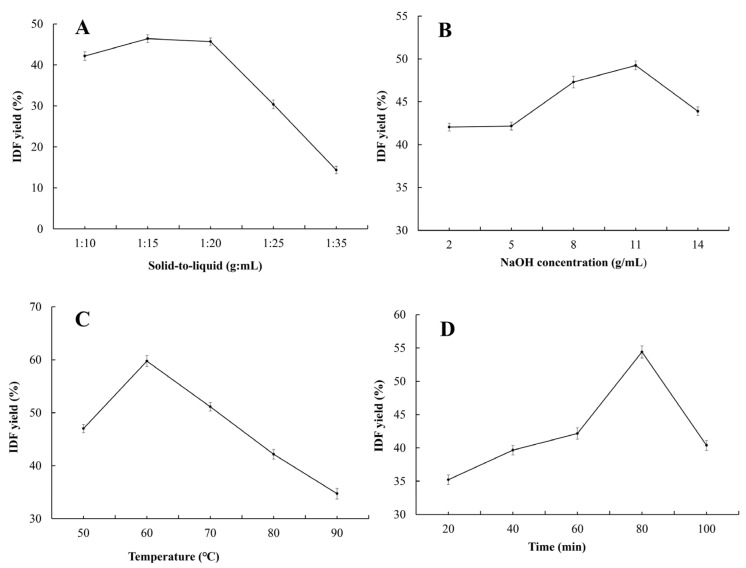
The effect of alkaline factors on the yield of IDF from PP: (**A**) solid-to-liquid ratio; (**B**) NaOH concentration; (**C**) temperature; and (**D**) time.

Figure 2A–D show the effects of single factors on IDF yield. The yield increased first and then decreased with the rise in temperature, NaOH concentration, extraction time, and solid–liquid ratio, with the optimal single-factor conditions at 60 °C, 11 g/L, 80 min, and 1:15 g/mL, respectively. Thus, 50/60/70 °C, 8/11/14 g/L, 60/80/100 min, and 1:10/1:15/1:20 g/mL were selected for orthogonal optimization.

#### 3.1.3. Orthogonal Experimental Optimization of IDF Extraction Conditions

The extraction of IDF primarily involves physical disruption of the cell wall and impurity removal, with a process response that may exhibit closer linearity. Therefore, orthogonal methods are employed for high-efficiency screening. Based on the single-factor experiments, extraction yield was chosen as the evaluation index. Four factors—extraction time, extraction temperature, NaOH concentration, and solid-to-liquid ratio—were selected for an L9(3^4^) orthogonal design, and the resulting data were analyzed. As shown in Table 3 and Table 4, the k values indicate that the order of influence on the extraction yield of IDF from PP is A (extraction temperature) > B (extraction time) > D (solid-to-liquid ratio) > C (NaOH concentration). The effects of A and B on the extraction yield are extremely significant. The optimal process parameters are A_2_B_2_C_3_D_3_, corresponding to an extraction time of 80 min, an extraction temperature of 60 °C, a NaOH concentration of 14 g/L, and a solid-to-liquid ratio of 1:20 g/mL. Under these conditions, triplicate verification experiments yielded an average extraction of 62.18 ± 1.25%, with good repeatability and strong stability. This finding aligns with reports in similar research. For instance, Wang et al. [34] also reported that alkaline extraction yielded a higher amount of water-insoluble dietary fiber (62.80%) from pineapple pomace compared to acid extraction.

### 3.2. Optimization of Extraction Conditions for SDF from PP

#### 3.2.1. Extraction Method Investigation

**Figure 3:**
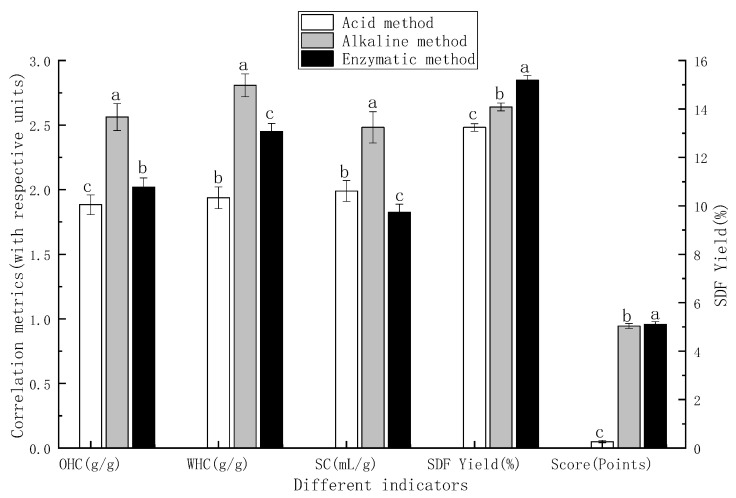
Comparison of different extraction methods for SDF. Note: The right vertical axis represents the values of extraction yield, while the left vertical axis represents those of OHC, WHC, SC, and the score. Different letters denote significant differences (*p* < 0.05).

A comparative analysis of the effects of acid, alkaline, and enzymatic methods on the yield of SDF from PP is illustrated in Figure 3. According to the weight distribution, with the extraction rate accounting for 80% and other functional indicators collectively accounting for 20%, the scores of the acid method, alkali method, and enzymatic method are 0.0502, 0.9446, and 0.9578, respectively. Therefore, the enzymatic method is selected as the extraction method for soluble dietary fiber (SDF) for further optimization.

#### 3.2.2. Single Factor Optimization of SDF Extraction Method

**Figure 4:**
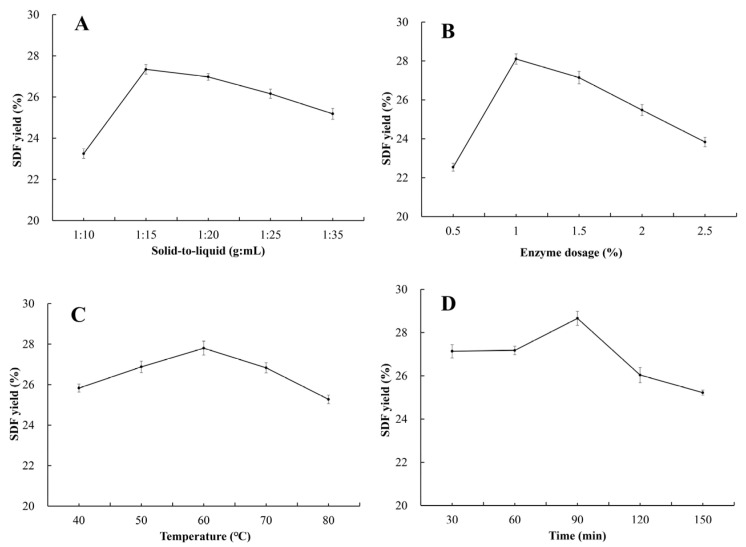
The effect of single-factor enzymatic hydrolysis on the yield of SDF from PP: (**A**) solid-to-liquid ratio; (**B**) enzyme dosage; (**C**) temperature; and (**D**) time. Note: Data are expressed as mean ± standard deviation (mean ± SD) with n = 3.

Figure 4A–D show the effects of single factors on enzymatic SDF yield. The yield of SDF increased first and then decreased with the increase in solid-to-liquid ratio, enzyme dosage, temperature, and time, with the optimal single-factor conditions at 1:15, 1.0%, 60 °C, and 90 min, respectively. These levels were set as the center points for subsequent RSM optimization.

#### 3.2.3. Response Surface Optimization of SDF Extraction Conditions

The extraction of SDF is usually dependent on precise enzymatic hydrolysis or chemical transformation. Enzyme activity is highly sensitive to temperature and pH, and there may be significant nonlinear interactions among these factors. Therefore, it is more scientific to use response surface methodology for optimization. Three levels of response surface analysis were conducted using Box–Behnken experiments, with four main factors selected: solid-to-liquid ratio A, enzyme dosage B, temperature C, and time D. The response surface test results are shown in Table 5.

By employing Design-Expert 10.0.3 to analyze the experimental data, a second-order polynomial regression equation was established: Yield Y = 28.6341 + 0.0548167 A + 0.114808 B + 0.453092 C + 0.304933 D − 1.27938 AB + 1.18967 AC + 0.73025 AD − 1.57263 BC + 0.186875 BD − 0.227725 CD − 1.7 A^2^ − 2.32531 B^2^ − 2.09102 C^2^ − 3.0596 D^2^. The corresponding analysis of variance is presented in Table 6. In this context, the F-value is used to evaluate the significance of each variable’s influence on the response: the larger the F-value, the greater the significance. A model is considered statistically significant when the associated probability *p* < 0.05. According to Table 6, the impact of the process parameters on yield follows the order C > D > B > A, i.e., temperature (C) exerts the strongest influence, followed by time (D), enzyme dosage (B), and solid-to-liquid ratio (A). The significance of the model terms can be attributed to the underlying physicochemical nature of the extraction process. The dominant linear effect of temperature (C) follows the Arrhenius law. Significant quadratic terms confirm optimal points beyond which inhibition (e.g., enzyme denaturation) occurs. The critical interaction effects reveal that mass transfer (governed by factors like solid-to-liquid ratio A, per Fick’s law) and reaction kinetics (governed by enzyme dosage B and temperature C) are coupled. Thus, the statistical model captures the synergy and constraints of this diffusion-influenced enzymatic system.

It is noteworthy that while the main effects of A and B are not significant, their quadratic terms and interaction term are highly significant. This indicates that the optimal solid-to-liquid ratio is dependent on the enzyme dosage, and that the strong interaction may effectively mask the statistical significance of the main effects, rendering their respective linear contributions less meaningful [35].

The coefficient of determination R^2^ for the model is 0.9773, indicating a high level of significance. The adjusted coefficient Adj R^2^ is 0.9545, explaining 95.45% of the variation in the response, and is close to the predicted R^2^, confirming that the model fits the actual data well and possesses practical predictive value. Consequently, this model can be reliably employed to analyze and predict the optimal extraction conditions for maximizing yield.

**Figure 5:**
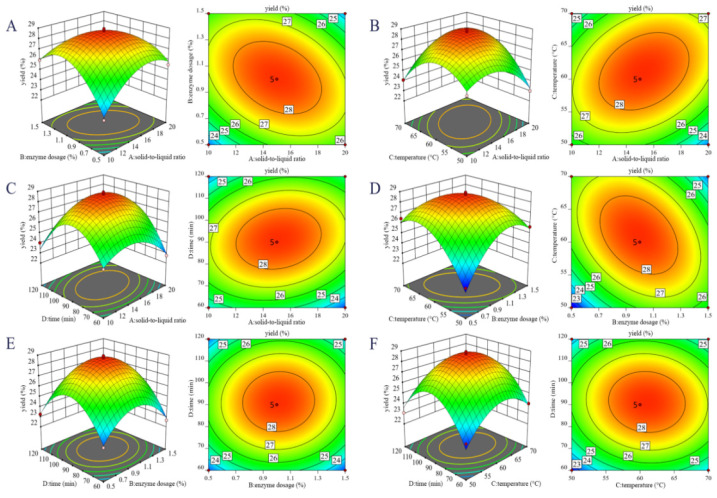
The effect of the interaction between different factors in enzymatic hydrolysis on the yield of PP SDF: (**A**) solid-to-liquid ratio and enzyme dosage; (**B**) solid-to-liquid ratio and temperature; (**C**) solid-to-liquid ratio and extraction time; (**D**) enzyme dosage and temperature; (**E**) enzyme dosage and time; and (**F**) temperature and time.

Interactive effects of different process conditions on the yield are illustrated in Figure 5A–F. Figure 5A shows that the interaction between solid-to-liquid ratio and enzyme dosage produces a parabolic surface with a large vertical span and markedly elliptical contour lines, indicating a significant interaction effect. Yield first increases and then decreases with rising levels of both variables. Moderate conditions, solid-to-liquid ratio 1:12~1:18 (g/mL), and enzyme dosage 0.95~1.05% give the highest product yield. In Figure 5B, the interaction surface for the solid-to-liquid ratio and temperature also exhibits an initial rise followed by a decline. Temperature has the steeper gradient, confirming its greater influence than the solid-to-liquid ratio. Optimal yield under these two factors is obtained at a solid-to-liquid ratio of 12:1~18:1 and a temperature of 55~65 °C. Figure 5C demonstrates that yield follows a similar trend with solid-to-liquid ratio and extraction time, but the time effect is more pronounced. The preferred range is a solid-to-liquid ratio of 12:1~18:1 and an extraction time of 80~100 min. Figure 5D reveals a pronounced interaction between enzyme dosage and temperature: the 3D surface has a large vertical span and elliptical contours. When enzyme dosage is below 1.0%, yield is positively correlated with dosage; above 1.0%, the trend reverses. The critical optimum is therefore ~1.0% enzyme dosage and 55~65 °C. Figure 5E presents a parabolic surface for enzyme dosage and time; yields are maximized at enzyme dosage 0.95~1.05% and time 80–100 min. Finally, Figure 5F shows nearly circular contours for the temperature and time interaction, implying this interaction is not significant. Yield peaks again and then declines as both variables rise, with the optimal interval being 55~65 °C and 80~100 min.

Combining all interactions and maximizing yield with Design-Expert 10.0.3, the software predicted the following optimal conditions: solid-to-liquid ratio 1:15.510 (g/mL), enzyme dosage 0.973%, temperature 61.543 °C, and time 91.622 min, with a predicted yield of 28.679%. To ensure practical feasibility, three replicate experiments were conducted at rounded settings: solid-to-liquid ratio 1:15.5, enzyme dosage 1.0%, temperature 61.5 °C, and time 92 min. The average experimental yield was (29.30% ± 0.15), closely matching the model prediction and confirming the validity and practicality of the response-surface-based optimization.

#### 3.2.4. Residual Analysis and Model Validation

**Figure 6:**
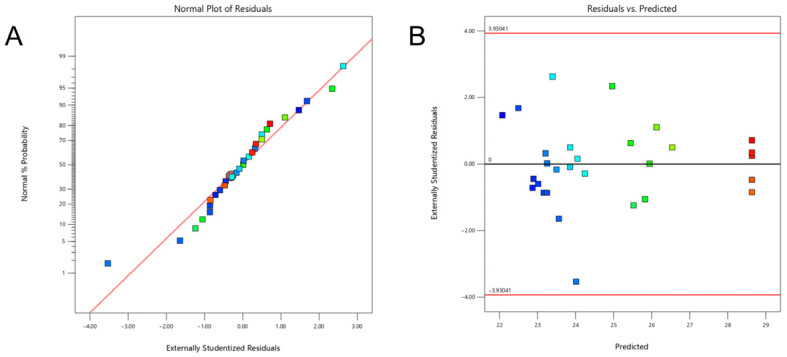
(**A**) Normal probability plot model; (**B**) residuals versus predicted values plot of the response surface.

To ensure the validity of the analysis of variance (ANOVA) for the response surface model, residual diagnostics were performed. As shown in the normal probability plot of residuals (Figure 6A), the data points generally followed a straight line, indicating that the residuals approximately conformed to a normal distribution. Furthermore, the plot of residuals versus predicted values (Figure 6B) revealed a random scatter of points without obvious patterns or trends, confirming the homogeneity of variance and the absence of significant outliers. The response surface model has been verified to be applicable for optimizing the extraction process of SDF.

### 3.3. PP IDF Activity Analysis

As shown in Table 7, the OHC of PP IDF and PP are 2.15 g/g and 1.48 g/g, respectively. Compared with the PP, the OHC of the alkali-prepared PP IDF is 1.45-fold higher. The WHC is 5.39 g/g for PP IDF and 2.69 g/g for the PP, indicating a two-fold increase, so the WHC of the alkali-prepared fiber is significantly superior. The SC is 2.91 mL/g for PP IDF and 1.98 mL/g for the PP, representing a 1.46-fold improvement. These results demonstrate that the alkali-prepared PP IDF exhibits better activity than the PP.

### 3.4. Activity Analysis of Modified PP SDF

The enhanced functional properties (WHC, OHC, CAC, GAC, CEC) of the modified SDF, as measured in vitro, serve as standard initial screening tools for evaluating the techno-functional potential of dietary fibers. These properties are linked to established physiological mechanisms; for example, high water- and oil-holding capacities contribute to increased fecal bulk and satiety [31,36]; cholesterol and glucose adsorption capacities are indicators of the potential to inhibit intestinal absorption of these nutrients, which may support blood lipid and glucose management [37]; and cation exchange capacity is associated with bile acid binding, a process relevant to cholesterol metabolism [38].

PP SDF was modified using physical, biological, and chemical methods. The resulting functional properties are summarized in Table 8. Compared with the unmodified SDF, all modified samples exhibited marked improvements in WHC, OHC, SC, CAC, GAC, and CEC. First, compared with the unmodified (native field) sample, all three modification methods (physical, biological, and chemical) significantly improved the functional properties of SDF (all *p* < 0.05).

Physical modification primarily disrupts the compact cell wall matrix, increasing fiber porosity, surface area, and exposure of hydrophilic groups, thereby improving its physical entrapment and binding capacity for water, oil, and molecules [39]. The physically modified SDF exhibited the highest SC (3.98 ± 0.05 g/mL).

Chemical treatment likely introduced or exposed certain functional groups, thereby enhancing the fiber’s glucose adsorption capacity [40]. The chemically modified SDF demonstrated the highest GAC (16.89 ± 0.13 mmol/g). But it might introduce chemical reagent residues [12].

However, the Biological method showed the most remarkable enhancement in most indicators, especially in SC (2.76 ± 0.05 g/mL), CAC (7.68 ± 0.10 mg/g), GAC (16.66 ± 0.16 mmol/g), and CEC (3.28 ± 0.06 mmol/g), which were all the highest values among the four groups, which is consistent with the research results of Li et al. [25] on corn bran SDF, It may be because microbial fermentation can enhance adsorption capacity by degrading fibrous macromolecules and increasing surface-active sites [41].

Consequently, biological modification emerges as the preferred method for producing high-quality, food-grade functional SDF ingredients.

A multi-factor comprehensive evaluation was then applied to quantitatively compare the SDF before and after modification. All data were normalized to a 0–1 range to ensure reliable comparisons: each parameter was divided by its maximum value within the group. The normalized results are presented in Table 9. According to the processed data, the physically modified SDF showed the highest WHC, whereas the biologically modified SDF exhibited the best OHC, CAC, and CEC. The chemically modified SDF demonstrated the greatest SC and GAC. Overall, the biologically modified SDF achieved the highest composite score of 5.66, indicating that it possesses the superior quality among the three modification approaches. This is consistent with the findings of Ren et al. [42] on rice bran dietary fiber. They discovered that fermentation (biological) modification increased the specific surface area of dietary fiber, exposing more enzyme-binding sites and active functional groups, thereby enhancing physiological activity.

## 4. Conclusions

This study systematically optimized the extraction processes for both SDF and IDF from PP using the DOE method. Modification strategies for SDF were explored, and the bioactivities of the resulting fibers were comprehensively evaluated. Alkaline extraction proved optimal for IDF; orthogonal testing identified the best parameters as a solid-to-liquid ratio of 1:20 g/mL, 60 °C, 14 g/L NaOH, and 80 min, achieving an extraction yield of 62.18%, significantly higher than acid or enzymatic methods. Enzymatic extraction was superior for SDF; response surface optimization of SDF yielded 29.3% under a 1:15.5 solid-to-liquid ratio, 1.0% enzyme dosage, 61.5 °C, and 92 min. Modified SDF exhibited markedly enhanced functional properties. Physical modification most effectively boosted SC (3.98 ± 0.05 g/mL). Chemical modification most effectively boosted GAC (16.89 mmol/g). Biological modification achieved the highest comprehensive score (5.66), indicating the most pronounced improvements, particularly in OHC, CAC, and CEC. These results provide efficient, low-cost extraction methods for PP valorization. Modified SDF demonstrates significant potential for application in functional foods and nutraceuticals.

It should be emphasized that further investigation into the corresponding structural and compositional changes by scanning electron microscope (SEM), mass spectrometry (MS), or nuclear magnetic resonance (NMR) is required to elucidate the underlying structure-function relationships. Additionally, to validate the proposed physiological benefits associated with these enhanced properties, conducting in vivo studies (animal experiments or clinical trials) constitutes a critical step for translating the research findings into practical health applications.

## Figures and Tables

**Table 1:** Factor and level design for the extraction process of IDF from PP.

Level	Factor			
	Extraction Temperature (°C)	Extraction Time (min)	Sodium Hydroxide Concentration (g/L)	Solid–Liquid Ratio (g/mL)
1	50	60	8	1:10
2	60	80	11	1:15
3	70	100	14	1:20

**Table 2:** Coded and actual levels of the independent variables in the Box–Behnken design.

Coded Levels	A Solid-to-Liquid Ratio (g/mL)	B Enzyme Dosage (%)	C Temperature (°C)	D Time (min)
−1	1:10	0.5	50	60
0	1:15	1.0	60	90
1	1:20	1.5	70	120

**Table 3:** Orthogonal experiment analysis result.

Number	Factor				Result
	A Temperature (°C)	B Time (min)	C NaOH Concentration (g/L)	D Solid-to-Liquid Ratio (g/mL)	Yield(%)
1	1	1	1	1	47.26 ± 0.60
2	1	2	2	2	52.15 ± 1.07
3	1	3	3	3	49.39 ± 1.03
4	2	1	2	3	56.54 ± 1.00
5	2	2	3	1	60.13 ± 1.25
6	2	3	1	2	53.64 ± 0.92
7	3	1	3	2	48.32 ± 0.79
8	3	2	1	3	53.46 ± 0.85
9	3	3	2	1	48.28 ± 0.79
k1	49.600	50.707	51.453	51.890	
k2	56.770	55.247	52.323	51.370	
k3	50.202	50.437	52.613	53.130	
R	7.170	4.810	1.160	1.760	
Optimal condition	A2	B2	C3	D3	
Influence factor	A > B > D > C				

**Table 4:** Orthogonal experimental analysis of variance.

Source of Variance	Sum of Squares	Degrees of Freedom	Mean Square	F-Value	Significance
Revised model	444.381	8	55.548	63.233	**
Nodal increment	73,373.496	1	73,373.496	83,525.565	**
A	291.57	2	145.785	165.956	**
B	131.563	2	65.782	74.883	**
C	6.531	2	3.265	3.717	
D	14.717	2	7.358	8.377	
Error	15.812	18	0.878		
SUM	73,833.69	27			
Adjusted SUM	460.93	26			

Note: ** indicates extremely significant difference (*p* < 0.01).

**Table 5:** Response surface experimental design and results.

Number	A Solid-to-Liquid Ratio (g/mL)	B Enzyme Dosage (%)	C Temperature (°C)	D Time (min)	Y SDF Yield (%)
1	−1	−1	0	0	22.91 ± 0.21
2	0	0	1	1	23.25 ± 0.16
3	1	0	0	1	25.56 ± 0.22
4	1	0	0	−1	22.76 ± 0.18
5	0	0	0	0	28.29 ± 0.28
6	0	0	−1	1	23.11 ± 0.17
7	−1	1	0	0	25.95 ± 0.26
8	0	0	0	0	28.74 ± 0.21
9	−1	0	1	0	24.10 ± 0.15
10	0	0	1	−1	24.01 ± 0.14
11	1	1	0	0	23.45 ± 0.18
12	0	0	0	0	28.44 ± 0.32
13	1	0	−1	0	23.00 ± 0.20
14	0	−1	0	1	23.26 ± 0.25
15	−1	0	0	−1	24.16 ± 0.17
16	0	−1	−1	0	22.49 ± 0.24
17	0	−1	0	−1	22.84 ± 0.17
18	0	1	1	0	23.31 ± 0.23
19	0	−1	1	0	26.45 ± 0.27
20	1	0	1	0	26.69 ± 0.22
21	1	−1	0	0	25.52 ± 0.18
22	0	0	0	0	28.93 ± 0.34
23	0	0	−1	−1	22.96 ± 0.18
24	0	1	0	1	23.83 ± 0.16
25	−1	0	−1	0	25.17 ± 0.24
26	0	1	−1	0	25.64 ± 0.25
27	0	0	0	0	28.78 ± 0.33
28	−1	0	0	1	24.04 ± 0.21
29	0	1	0	−1	22.66 ± 0.18

**Table 6:** Analysis of variance results of response yield fitting the regression equation.

Source of Variance	Sum of Squares	Degrees of Freedom	Mean Square	F-Value	*p*-Value	Significance
Regression model	123.33	14	8.81	43	<0.0001	**
A	0.0352	1	0.0352	0.1718	0.6848	
B	0.1564	1	0.1564	0.7634	0.397	
C	2.47	1	2.47	12.04	0.0038	**
D	1.12	1	1.12	5.45	0.035	*
AB	6.53	1	6.53	31.86	<0.0001	**
AC	5.66	1	5.66	27.65	0.0001	**
AD	2.13	1	2.13	10.4	0.0061	**
BC	9.89	1	9.89	48.28	<0.0001	**
BD	0.1406	1	0.1406	0.6864	0.4213	
CD	0.207	1	0.207	1.01	0.3318	
A2	18.77	1	18.77	91.6	<0.0001	**
B2	35.09	1	35.09	171.28	<0.0001	**
C2	28.39	1	28.39	138.58	<0.0001	**
D2	60.72	1	60.72	296.39	<0.0001	**
Residual	2.87	14	0.2049			
Omission item	2.59	10	0.2592	3.76	0.107	
Pure error	0.2761	4	0.069			
Sum	126.2	28				
R2	0.9773					
Adj R2	0.9545					
Pred R2	0.8783					

Note: ** indicates extremely significant difference (*p* < 0.01); * indicates significant difference (*p* < 0.05).

**Table 7:** Analysis results of PP IDF activity.

Sample	WHC (g/g)	OHC (g/g)	SC (g/mL)
PP	2.69 ± 0.06 ^b^	1.48 ± 0.05 ^b^	1.98 ± 0.06 ^b^
PP IDF	5.39 ± 0.07 ^a^	2.15 ± 0.04 ^a^	2.91 ± 0.08 ^a^

Note: Data are expressed as mean ± standard deviation (mean ± SD) with *n* = 3 (three parallel experiments); different lowercase letters after data in the same column indicate significant differences between groups (*p* < 0.05, Duncan’s multiple comparison method).

**Table 8:** Activity analysis results of SDF before and after modification.

Method	WHC (g/g)	OHC (g/g)	SC (g/mL)	CAC (mg/g)	GAC (mmol/g)	CEC (mmol/g)
Unmodified	2.45 ± 0.06 ^d^	2.02 ± 0.07 ^d^	1.83 ± 0.06 ^d^	2.99 ± 0.08 ^d^	6.24 ± 0.09 ^c^	0.47 ± 0.01 ^d^
Physical	3.70 ± 0.07 ^c^	3.89 ± 0.08 ^b^	3.98 ± 0.05 ^a^	6.38 ± 0.09 ^b^	13.77 ± 0.15 ^b^	2.03 ± 0.02 ^c^
Biological	5.58 ± 0.05 ^a^	4.38 ± 0.06 ^a^	2.76 ± 0.05 ^c^	7.68 ± 0.10 ^a^	16.66 ± 0.16 ^a^	3.28 ± 0.05 ^a^
Chemical	4.74 ± 0.07 ^b^	3.10 ± 0.04 ^c^	3.54 ± 0.08 ^b^	5.03 ± 0.07 ^c^	16.89 ± 0.13 ^a^	2.28 ± 0.06 ^b^

Note: Data are expressed as mean ± standard deviation (mean ± SD) with n = 3; different lowercase letters after data in the same column indicate significant differences between groups (*p* < 0.05, Duncan’s multiple comparison method); WHC: water-holding capacity, OHC: oil-holding capacity, SC: swelling capacity, CAC: cholesterol adsorption capacity, GAC: glucose adsorption capacity, CEC: cation-exchange capacity.

**Table 9:** Homogenization results of PP SDF activity analysis before and after modification.

Method	WHC	OHC	SC	CAC	GAC	CEC	Score
Unmodified	0.43	0.46	0.40	0.39	0.37	0.14	2.19
Physical	1.00	0.89	0.88	0.83	0.82	0.62	5.03
Biological	0.98	1.00	0.69	1.00	0.99	1.00	5.66
Chemical	0.83	0.71	1.00	0.65	1.00	0.70	4.89

## Data Availability

The original contributions presented in this study are included in the article. Further inquiries can be directed to the corresponding author.
